# The Time Course of Deafness and Retinal Degeneration in a Kunming Mouse Model for Usher Syndrome

**DOI:** 10.1371/journal.pone.0155619

**Published:** 2016-05-17

**Authors:** Lu Yao, Lei Zhang, Lin-Song Qi, Wei Liu, Jing An, Bin Wang, Jun-Hui Xue, Zuo-Ming Zhang

**Affiliations:** 1 Department of Clinical Aerospace Medicine, Fourth Military Medical University, 169 West Changle Road, Xi'an, China; 2 Department of Physical Examination, Air Force General Hospital, 30 Fucheng Road, Beijing, China; 3 Medical Experiment Center, Shaanxi University of Chinese Medicine, Shiji Ave., Xi'an-Xianyang New Ecomic Zone, 712046, Xi'an, China; National Eye Institute, UNITED STATES

## Abstract

Usher syndrome is a group of autosomal recessive diseases characterized by congenital deafness and retinitis pigmentosa. In a mouse model for Usher syndrome, KM^ush/ush^_,_ discovered in our laboratory, we measured the phenotypes, characterized the architecture and morphology of the retina, and quantified the level of expression of *pde6b* and *ush2a* between postnatal (P) days 7, and 56. Electroretinograms and auditory brainstem response were used to measure visual and auditory phenotypes. Fundus photography and light microscopy were used to measure the architecture and morphology of the retina. Quantitative real-time PCR was used to measure the expression levels of mRNA. KM^ush/ush^ mice had low amplitudes and no obvious waveforms of Electroretinograms after P14 compared with controls. Thresholds of auditory brainstem response in our model were higher than those of controls after P14. By P21, the retinal vessels of KM^ush/ush^ mice were attenuated and their optic discs had a waxy pallor. The retinas of KM^ush/ush^ mice atrophied and the choroidal vessels were clearly visible. Notably, the architecture of each retinal layer was not different as compared with control mice at P7, while the outer nuclear layer (ONL) and other retinal layers of KM^ush/ush^ mice were attenuated significantly between P14 and P21. ONL cells were barely seen in KM^ush/ush^ mice at P56. As compared with control mice, the expression of *pde6b* and *ush2a* in KM^ush/ush^ mice declined significantly after P7. This study is a first step toward characterizing the progression of disease in our mouse model. Future studies using this model may provide insights about the etiology of the disease and the relationships between genotypes and phenotypes providing a valuable resource that could contribute to the foundation of knowledge necessary to develop therapies to prevent the retinal degeneration in patients with Usher Syndrome.

## Introduction

Retinitis pigmentosa (RP) is a very common form of inherited retinal degeneration characterized by a gradual degeneration of rod and cone photoreceptors resulting in decreased dark adaptation and night blindness, loss of mid-peripheral visual field and eventually central vision loss [1]. The prevalence of RP is 1/4000 worldwide [2–4]. RP is a group of highly heterogeneous diseases with variable phenotypes involving a multiplicity of genes and mutations [5]. At least 100 genes and more than 4000 mutations are related to RP [6]. RP is classified as autosomal dominant (30–40%), autosomal recessive (50–60%) and X-linked (5–15%) [7]. In some cases, RP can also occur in the absence of a family history or in mitochondrial and digenic inheritance [8]. RP that develops with many diseases is defined as syndromic RP [9].

Usher syndrome (USH) is an autosomal recessive disorder characterized by combined RP and deafness, the most common form of syndromic RP. It is the most prevalent cause of hereditary deafness and blindness. Except for the manifestation of RP, USH can be divided into three clinical types according to the onsite time and the degree of deafness, and the occurrence of vestibular dysfunction. Type 1 USH (USH1) is characterized by profound deafness at birth and vestibular dysfunction [10]. Type 2 USH, USH2, is the most prevalent form of USH. Patients with USH2 suffer from moderate and non-progressive hearing loss and normal vestibular dysfunction [11]. Patients with type 3 USH, USH3, have gradual hearing loss with or without vestibular dysfunction [12]. Similar to RP, USH is also genetically and clinically heterogeneous. USH is associated with 16 loci among 13 genes [12, 13]. Mutations in *ush2a* can lead to USH2 or non-syndromic RP [14].

To understand the etiology of inherited retinal disease, numerous studies have been conducted based on animal models and clinical studies, yet genes and proteins involved in RP or USH are not fully known. For RP, a large proportion of clinical features overlap between different types, and even the same mutation may cause different clinical symptoms [8, 15]. Although several therapeutic strategies can slow the development of RP, few therapies can rescue or reverse photoreceptor loss [16].

Proteins encoded by genes related to USH often form complexes and function cooperatively [17]. However, our current knowledge about genes and proteins related to USH are still incomplete, especially related to their retinal function. There is no cure to ameliorate visual and auditory symptoms at the same time.

Mice models are excellent for mimicking diseases in order to investigate the molecular basis related to the efficacy of therapies. To date, there are many RP or USH mouse models. Most of them are transgenic or chemically induced, which may not simulate human diseases. For instance, most USH mouse models exhibit an indistinctive ocular phenotype [18]. Through electroretinogram (ERG) screening, our laboratory discovered a strain of mice with spontaneous RP derived from Kunming mice. The strain has been inbred to 26 generations with a stable phenotype in autosomal recessive heritance, which we designated as KM^ush/ush^. In a preliminary study, our mouse model expressed lower levels of *pde6b* and *ush2a* mRNA. In the present study we examined the development of visual and auditory phenotypes as well as the gene expression of *pde6b* and *ush2a*, laying the foundation for future gene sequencing studies and studies to elucidate the etiology of the disease with the ultimate goal of finding a cure.

## Materials and Methods

### Animals

KM^ush/ush^ mice were obtained from our departmental pathogen free animal lab. The mice were of Kunming background, and expanded using a single colony through 26 generations of sibling matings. The wild-type Kunming mice were obtained from the Laboratory Animal Center of the Fourth Military Medical University. All KM^ush/ush^ mice were compared with appropriately age- and strain-matched control mice. All animals were kept on a 12 h light/dark cycle, with food and water available *ad libitum* throughout the studies. All animal experiments were performed in accordance with the ARVO (The Association for Research in Vision and Ophthalmology) statement for the use of animals in ophthalmic and vision research and were approved by the Animal Care and Use Committee of the Fourth Military Medical University.

### ERG

ERG recordings were carried out at postnatal day (P) 14 (n = 8), P21 (n = 6), and P56 (n = 6). After overnight dark adaption, KM^ush/ush^ and control mice were deeply anesthetized with 3 mL/kg intraperitoneal injection of 1% sodium pentobarbital (Sigma, St Louis, MO, USA, P3761) and 50 μL sumianxin II (Jilin Shengda Animal Pharmaceutical Co., Ltd., Jilin, China). The pupils were dilated with 0.5% tropicamide-phenylephrine ophthalmic solution (Shenyang Xingqi, Pharmaceutical Co., Ltd., Shenyang, China). The active electrode, a silver-chloride electrode loop encased in a layer of 1% methylcellulose, was placed on the cornea. The reference electrode and ground electrode were inserted beneath the skin of the cheek around the tested eye and tail separately. Full-field (Ganzfeld) stimulation and a commercial system (RETI port; Roland Consult GmbH, Brandenburg, Germany) were applied to record ERGs with a band pass of 0.5–1000 Hz. All operations were conducted under a dim red light to maximize retinal sensitivity. Scotopic 0.01 cd.s.m^-2^, 3.0 cd.s.m^-2^, 3.0 cd.s.m^-2^ OPs ERG, and photopic 3.0 cd.s.m^-2^, 3.0 cd.s.m^-2^ Flicker ERG were recorded. Levofloxacin eye drops (Santen Pharmaceutical (China) Co., Ltd, Suzhou, China) were used three times a day after ERG testing to avoid infection.

### Auditory brainstem response (ABR)

ABR was performed on KM^ush/ush^ and age-matched control mice. Animals were anesthetized with 3 mL/kg intraperitoneal injection of 1% sodium pentobarbital (Sigma, St Louis MO, USA, P3761) and 50 μL sumianxin II (Jilin Shengda Animal Pharmaceutical Co., Ltd., Jilin, China). The active, reference and ground electrodes were placed subcutaneously in the vertex auricle of the tested ear and tail, respectively. The responses to click stimuli were collected at P30. A series of responses to 4, 8, 16, 24 and 32 kHz tone bursts were routinely presented to mice at P14 (n = 10), P21 (n = 10) and P56 (n = 12). Each stimuli was presented initially at 90 dB SPL (sound intensity level) and the SPL was progressively reduced by 5 dB to identify the threshold in which a recognized ABR waveform could be present and repeatable (if there were no detectable waveforms at 95 or greater than 95 dB SPL, the threshold was recorded as 90 dB SPL).

### Fundus photography

To image the fundus, KM^ush/ush^ and control mice were examined at P21. Animals were anesthetized with 3 ml/kg injection of 1% sodium pentobarbital (Sigma, St Louis, MO, USA, P3761) and 50 μL sumianxin II (Jilin Shengda Animal Pharmaceutical Co., Ltd., Jilin, China), and placed on a platform to keep their bodies fixed. The pupils were dilated with 0.5% tropicamide-phenylephrine ophthalmic solution (Shenyang Xingqi, Pharmaceutical Co., Ltd., Shenyang, China) and the corneas were covered with medical sodium hyaluronate gel (Bausch & Lomb Freda, Shandong, China) that touched the lens of a Micron III Retinal Imaging Microscope (Phoenix Research Laboratories, Pleasanton, CA). After the fundus images were taken, medical sodium hyaluronate gel was rinsed off the cornea with normal saline, and levofloxacin eye drops were used three times a day for three days to avoid infection.

### Measurement of retinal outer nuclear layer (ONL) thickness

Eyes of KM^ush/ush^ and control mice were enucleated rapidly at P7, 14, 21, and 56 after a lethal intraperitoneal injection of sodium pentobarbital. Tissues were paraffin embedded. Serial 3 μm thick sections were cut. For each eye, three sections that included the optic nerve were stained with hematoxylin and eosin. Images were taken using a digital imaging system (DP71, Olympus, Japan). Three vertical sections per eye were analyzed by counting the number of rows of ONL at the region 200 μm (central region) from the optic nerve on both sides.

### Total RNA isolation, reverse transcription-polymerase chain reaction (RT-PCR) and quantitative real-time PCR

Total RNA was extracted from retinas of KM^ush/ush^ and control mice at P7, 14, 21, 28 and 56 using the TRIzol reagent (Invitrogen, Grand Island, NY, USA). RNA (1 μg) from each sample was reverse transcribed into single-stranded complementary DNA (cDNA) using a Transcriptor First Stand cDNA Synthesis Kit (Roche, Mannheim, Germany) according to the manufacturer’s instructions. Amplification and quantification of cDNA were carried out in a 20 μL reaction mixture containing 10 μL 2× FastStart Universal SYBR Green Master Mix (Roche, Indianapolis, IN, USA), 1 μL cDNA, 1 μL primers and 8 μL ddH_2_O. The primers used in RT-PCR and quantitative real-time PCR were:

*Ush2a*:

5’-GTCACACATGCTTCCAGGTAATG-3’ (*forward*)

5’-GGGAACGGTAAATGGCTCTCTA-3’ (*reverse*)

*Pde6b*:

5’-GATCCAAGACAGTCCTCTCCAAG-3’ (*forward*)

5’–GACCACACGAAAGGAGATAGTCA-3’ (*reverse*)

All reactions were performed in triplicate. *β-actin* was used as an endogenous control. *Ush2a* and *Pde6b* expression levels were normalized to expression levels of *β-actin*. Then, the relative expression levels of *ush2a* and *Pde6b* were normalized to that of Kunming mice at P7 respectively.

### Statistical analysis

All data were expressed as mean ± standard deviations and analyzed using one-way analysis of variance (ANOVA) and Bonferroni tests for multiple comparisons. *P* < 0.05 was considered statistically significant.

## Results

### ERG analysis

**Fig 1:**
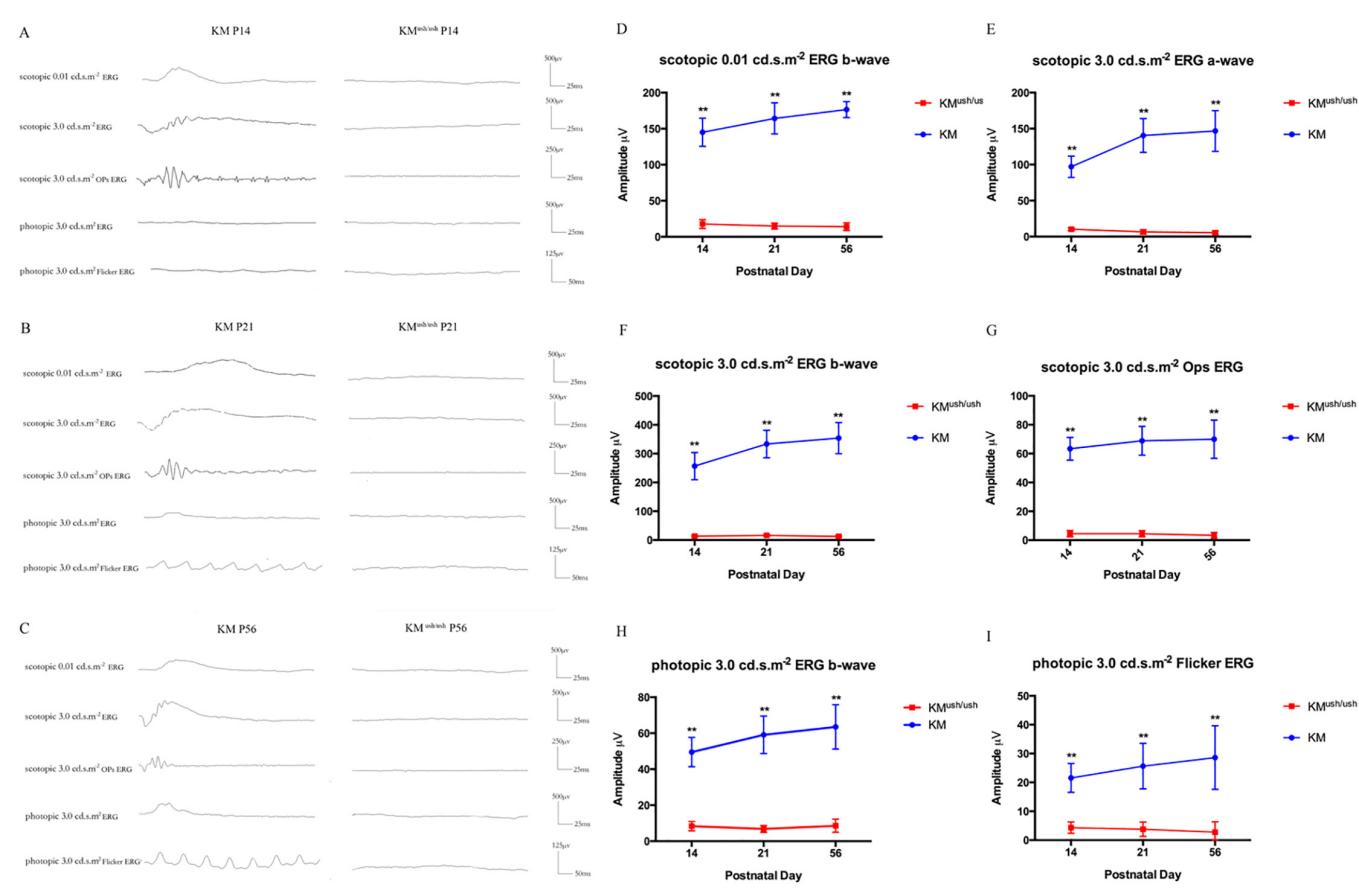
The visual function of KM^ush/ush^ mice decreased compared with controls. (A-C), Responses to scotopic 0.01 cd.s.m^-2^, 3.0 cd.s.m^-2^, 3.0 cd.s.m^-2^ OPs ERG, and photopic 3.0 cd.s.m^-2^, 3.0 cd.s.m^-2^ Flicker ERG at P14, P21 and P56 of KM^ush/ush^ and control mice showed that no obvious waveforms could be recorded of KM^ush/ush^ mice after P14. (D-I), Average amplitude of scotopic and photopic ERGs at P14 (n = 8), P21 (n = 6) and P56 (n = 6) of KM^ush/ush^ (red line) and control mice (blue line). Results were presented as means ± SD, Data were analyzed with one-way ANOVA. **, *P* < 0.001

Retinal function was measured using ERG to measure the summed electrical activity of all retinal cells. At P14, the time point at which the palpebral fissures open, no obvious waveforms could be recognized in KM^ush/ush^ mice and the mean amplitude of both scotopic and photopic ERGs declined significantly compared with controls (*P* < 0.01; Fig 1A and 1D-I). Similar changes were observed at P21 and P56 and no discernible waveforms could be observed in KM^ush/ush^ mice with scotopic and photopic ERG amplitude reduced remarkably (*P* < 0.01; Fig 1B-I). Compared with controls, ERG results indicated that KM^ush/ush^ mice exhibited retinal dysplasia and severe loss of functional rod and cone photoreceptors starting on P14.

### ABR analysis

**Fig 2:**
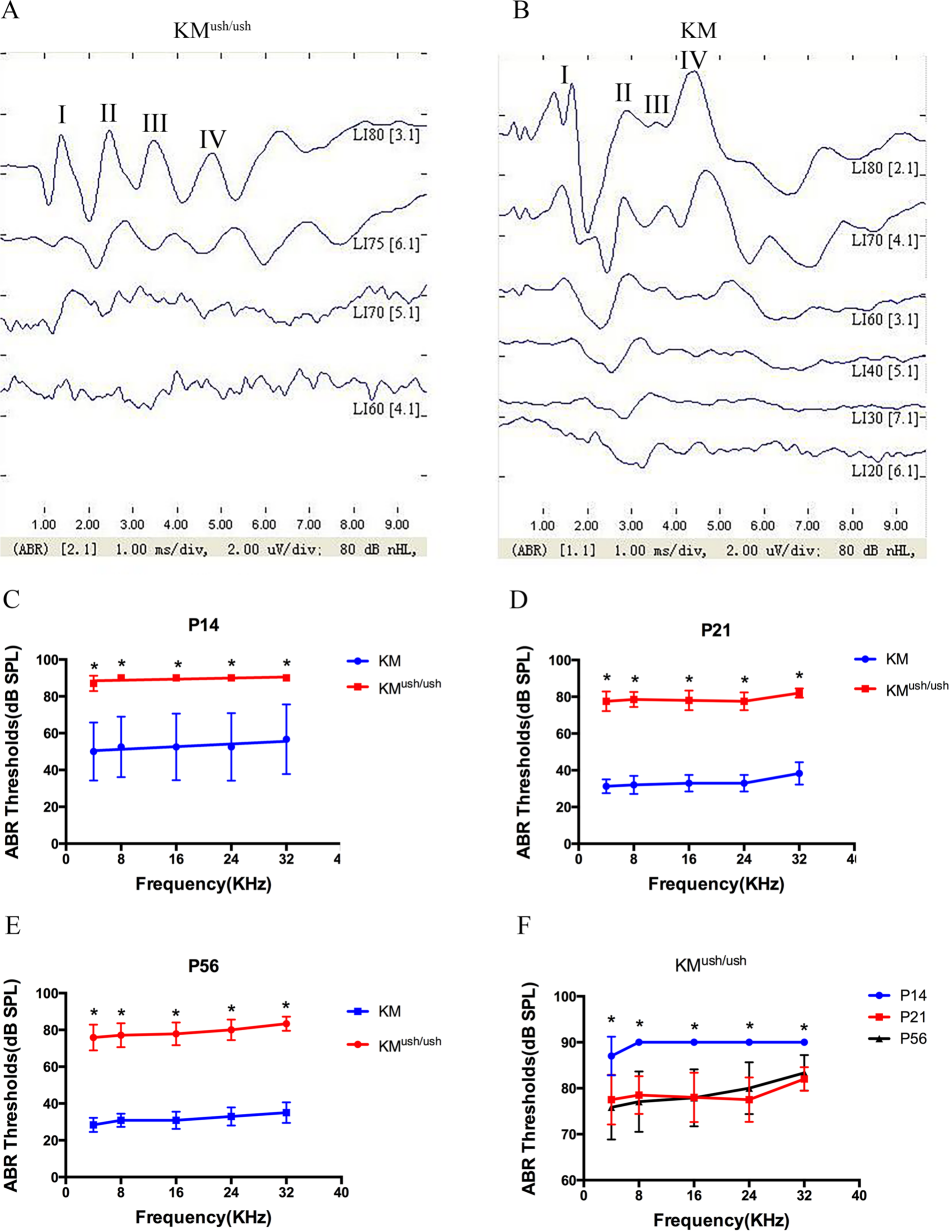
ABR tests for KM^ush/ush^ and age-matched controls. (A-B), Responses to click stimuli showed that the waveforms of ABR usually consist of 4 or 5 response peaks, labeled I, II, II, and IV respectively. The threshold of KM^ush/ush^ mouse at P30 was estimated as 75 dB SPL (A). The threshold of control mouse was estimated as 30 dB SPL (B). (C-E), Average thresholds of ABR tests to 4, 8, 16, 24 and 32 kHz tone bursts at P14 (n = 10), P21 (n = 10) and P56 (n = 12) of KM^ush/ush^ (red line) and control mice (blue line). (F), Average thresholds of KM^ush/ush^ mice at P14 (blue line) were higher than those of P21 (red line) and P56 (black line). Results were presented as means ± SD, Data were analyzed with one-way ANOVA. **, *P* < 0.001.

Initial evaluation of KM^ush/ush^ mice showed that the mRNA expression of *pde6b* and *ush2a* decreased simultaneously. To investigate these observations further, we performed ABR measurements to identify whether KM^ush/ush^ mice had auditory dysfunction when ush2a expression decreased. The waveforms of ABR usually consisted of 4 or 5 response peaks. At P30, responses to click stimuli showed that the obvious waveform could not be recognized when induced by a stimuli of 75 dB SPL, so the threshold of KM^ush/ush^ mice was estimated at 75 dB SPL (Fig 2A). Similarly, the threshold of control mice was estimated as 30 dB SPL (Fig 2B). At P14, ABR thresholds of KM^ush/ush^ mice responding to 4, 8, 16, 24 and 32 kHz tone bursts were 87.0 ± 1.3, 90.0 ± 0.0, 90.0 ± 0.0, 90.0 ± 0.0, 90.0 ± 0.0 dB SPL, respectively, were higher than controls (*P <* 0.01, Fig 2C). At P21, ABR thresholds to each stimuli were 77.5 ± 1.7, 78.5 ± 1.3, 78.0 ± 1.7, 77.5 ± 1.5, 82.0 ± 0.8 dB SPL, respectively (Fig 2D). At P56, ABR thresholds were 75.8 ± 2.0, 77.1 ± 1.9, 77.9 ± 1.8, 80.0 ± 1.6, 83.3 ± 1.1 dB SPL, respectively (Fig 2E). The average ABR thresholds were higher at P14 than P21 and 56 (*P* < 0.01, Fig 2F), indicating that auditory function improved to some degree with the development of the disease; however, KM^ush/ush^ mice exhibited severe hearing loss at birth.

### Morphological changes

**Fig 3:**
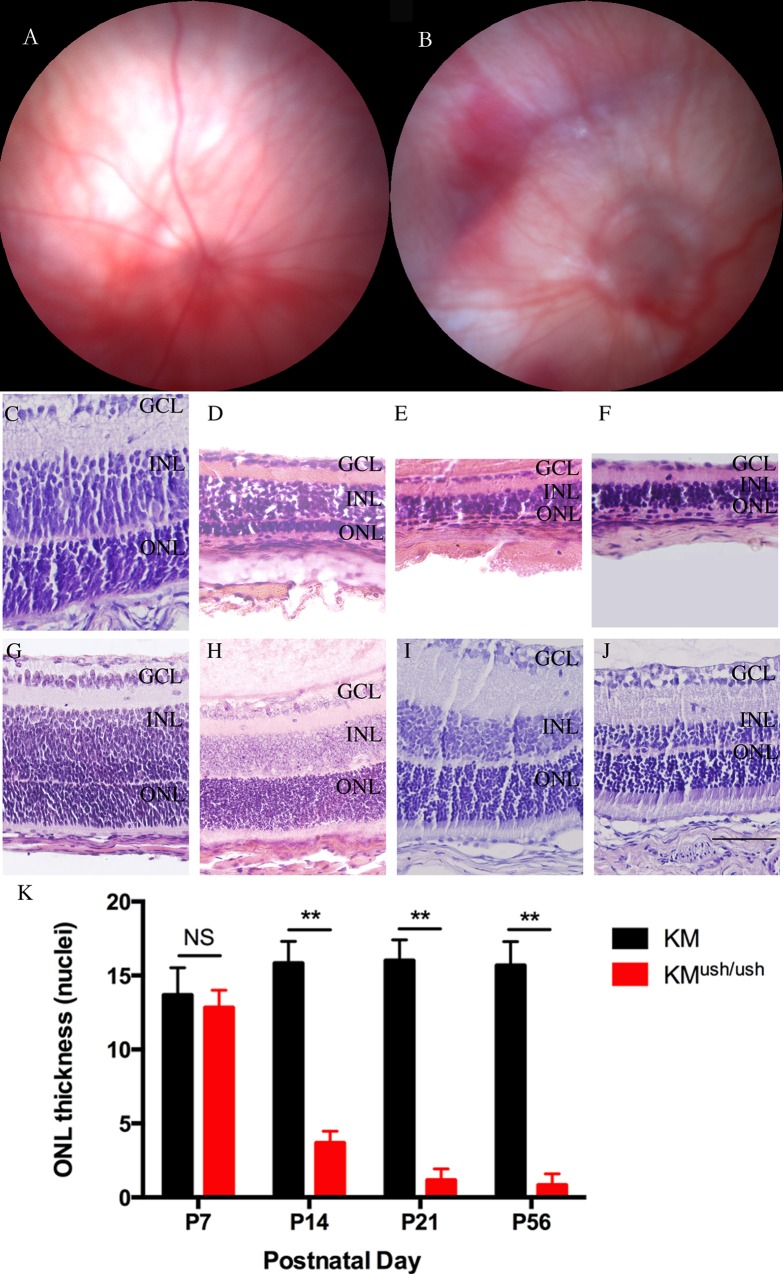
Morphology changes of fundus and retinas of KM^ush/ush^ and control mice. (A-B), Fundus photography performed at P21. KM^ush/ush^ mice (B) exhibited severe attenuation of retinal vessels and waxy pallor of the optic discs in comparison with controls (A). Atrophy of the retina and choroidal vessels were clearly visible. (C-J), Retinal sections of KM^ush/ush^ and control mice. (G-J), Retinas of control mice at P7 (G), P14 (H), P21 (I) and P56 (J). Retinas of Retinas of KM^ush/ush^ mice at P7 (C) had no differences in the thickness and structure of each retinal layer compared with control mice. At P14 (D), the ONL cells and other layers of retina including inner nuclear layer were attenuated. At P21 (E) and P56 (F) the ONL and the entire retinas became even thinner. (K), Plot of the thickness of ONL, measured as numbers of photoreceptor nuclei per column. Results are presented as means ± SD. Data were analyzed with one-way ANOVA. **, *P* < 0.001. ONL, outer nuclear layer; INL, inner nuclear layer; GCL, ganglion cell layer. Scale bar, 50 μm.

Fundus photography was performed at P21. Compared with controls (Fig 3A), KM^ush/ush^ mice exhibited severe attenuation of retinal vessels and optic discs with a waxy pallor. Moreover, atrophy of the choroidal vessels was clearly visible (Fig 3B). Since Kunming mice are albino, we could not observe the areas of retinal depigmentation. Morphological changes in the fundus showed early retinal degeneration in KM^ush/ush^ mice.

### Photoreceptor degeneration

To look for photoreceptor degeneration characteristic of Usher-associated retinitis pigmentosa, the thickness of retinal layer was compared with that of control mice at P7, P14, P21, and P56. At P7, no differences were found in the thickness and structure of each retinal layer between KM^ush/ush^ (12.8 ± 1.1 rows, n = 6, Fig 3C) and control mice (13.7 ± 1.9 rows, n = 6, *P* < 0.05, Fig 3G). However, at P14, the ONL cells were attenuated to only a few layers (3.6 ± 0.9 rows, n = 6, Fig 3D). In addition, other layers of the retina including the inner nuclear layer (INL), outer plexiform layer (OPL), and inner plexiform layer (IPL) also decayed severely when compared with controls (15.8 ± 1.5 rows, n = 6, *P* < 0.01, Fig 3H). At P21, there was only one layer of ONL cells remained and the entire retina became even thinner (1.2 ± 0.8 rows, n = 6, Fig 2E). At P56, ONL cells were barely seen and the thickness of KM^ush/ush^ mice retinas were approximately one fifth of those of controls (0.8 ± 0.8 rows, n = 6, Fig 3F).

### *Pde6b* and *ush2a* mRNA expressions

**Fig 4:**
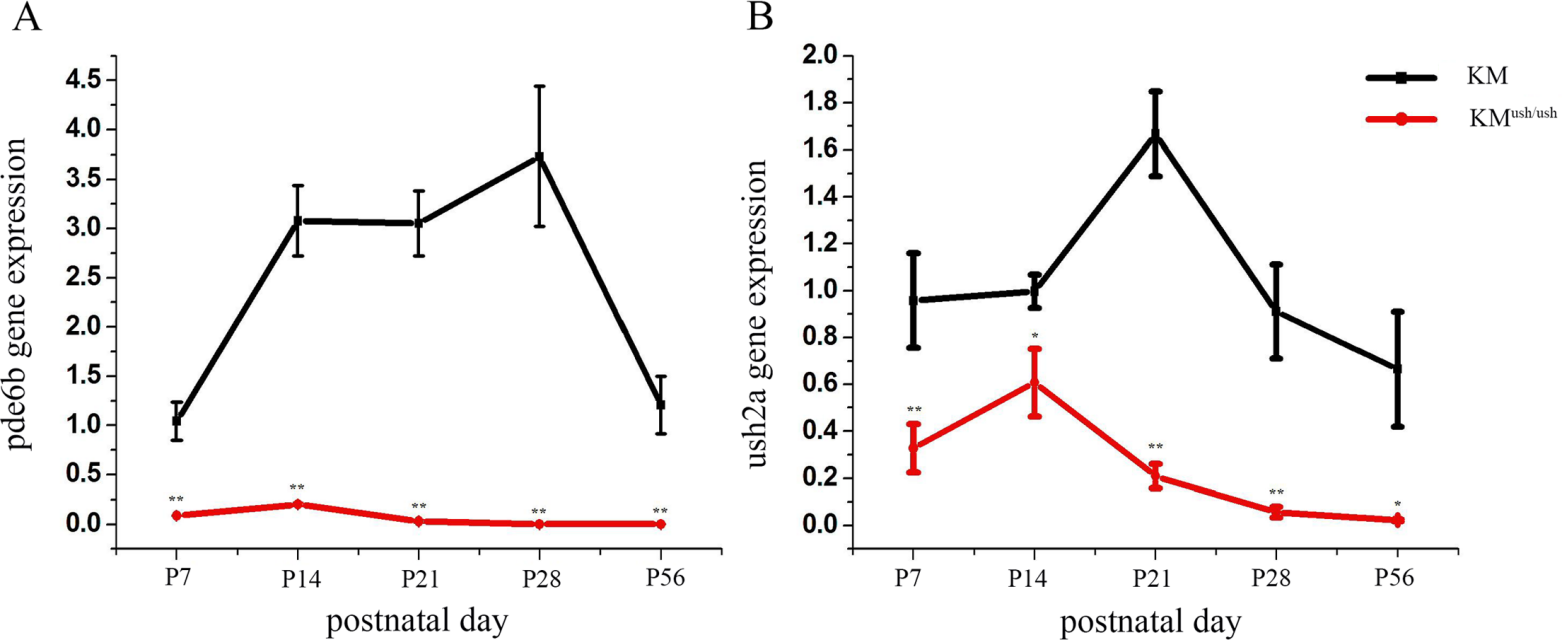
The expression of *pde6b* and *ush2a* in KM^ush/ush^ and control mice. The relative expression quantity of *pde6b* and *ush2a*. (A). The expression level of *pde6b* was much lower than controls at P7 but elevated in a narrow range to P14, and declined after P21. (B), The expression level of *ush2a* was lower compared with controls and demonstrated a similar trend as *pde6b*. Results are presented as means ± SD. Data were analyzed with one-way ANOVA. **, *P* < 0.001. *, *P* < 0.001.

We isolated the total mRNA of retinas from KM^ush/ush^ mice and controls at different postnatal days. Then, we assessed the expressions of *pde6b* and *ush2a* mRNA at P7, 14, 21, 28 and 56 using quantitative real time-PCR. The relative expression of *pde6b* for controls at P7, 14, 21, 28 and 56 were 1.04 ± 0.19, 3.07 ± 0.36, 3.05 ± 0.33, 3.73 ± 0.71, 1.21 ± 0.29, respectively (Fig 4A, black line), and the relative expression for KM^ush/ush^ mice was 0.090 ± 0.004, 0.20 ± 0.02, 0.03 ± 0.02, 0.0005 ± 0.0001, 0.0003 ± 1.96*10^−5^, respectively (Fig 4A, red line). Expression of *Ush2a* mRNA in controls was 0.96 ± 0.20, 0.97 ± 0.07, 1.67 ± 0.18, 0.91 ± 0.20, 0.67 ± 0.25 from P7 to P56 (Fig 4B, black line). For KM^ush/ush^ mice, the relative expression was 0.33 ± 0.10, 0.61 ± 0.14, 0.21 ± 0.05, 0.06 ± 0.02, 0.020 ± 0.004 (Fig 4B, red line). The level of mRNA expression of *pde6b* and *ush2a* in KM^ush/ush^ mice were lower compared with controls (Fig 4A and 4B, *P* < 0.05) and both exhibited a trend toward elevated expression from P7 to P14 within a narrow range followed by a decline to the baseline level. In controls, *Pde6b* expression increased from P7 to P28, and then decreased to the level of P7 at P56. A similar trend was noted for the expression of *ush2a*, which increased from P7 to P21 and then declined.

## Discussion

To understand the molecular mechanisms of USH disease induction and progression and to develop therapeutic strategies for the preservation of vision in patients with RP or USH, mutant (spontaneous and genetically engineered) mice models are the most widely used. In this study, we measured changes in ocular and auditory phenotypes and mRNA expression levels during the development of blindness and deafness in a mouse model discovered and inbred by our lab. It was important to characterize the time course of phenotypes because we needed to confirm that the deafness and retinal degeneration in this model was stable and to confirm the characteristics of the model similar to the approach used for rd1, the oldest and most-studied inherited model of retinal degeneration [19–21]. The time course of phenotypes as well as gene expression of *pde6b* and *ush2a* provides a foundation for future gene sequencing experiments.

The auditory phenotype of the KM^ush/ush^ mouse resembled those of USH2A human patients and the Ush2a^-/-^ mouse, which exhibit moderate, non-progressive hearing loss [18, 22]. While no visible ERG waveforms were evident in KM^ush/ush^ mice after P14, morphology changes in the fundus and retinas suggested early and rapid retinal degeneration. These ocular phenotypes resembled those of rd1 mice [23–25], which are characterized by early onset and severe lose of photoreceptors owing to a murine leukemia provirus insertion in intron 1 and a point mutation in exon 7 of *pde6b* gene mapped on mice chromosome 5 [26, 27]. So the early and rapid retinal degeneration of KM^ush/ush^ mice may be due to the mutation of rd1. In contrast, the photoreceptor degeneration in the *Ush2a* knockout mice is slowly progressive up to the age of 20 months where more than half of the ONL cells are lost and ERG amplitudes declined significantly [18]. Interestingly, none of the spontaneous mutations in Usher 1-related mouse models develop an overt photoreceptor degeneration [28–30] as found in Usher I patients, but both the induced and inherited models of Usher II exhibit retinal degeneration.

Since RP and USH are both genetically and clinically heterogeneous, more than one genome feature could exist in humans and mouse models. A case was reported in human that carried mutations in *pde6b* and *gpr98* genes (underlying USH2C) that increased the severity of the phenotypes compared with siblings who were homozygous for only one of the two genes [31]. In addition, a young patient with *pde6b* and *myo7a* gene mutations (underlying USH1B) presented earlier and more sever retinal degeneration than his older siblings with a homozygous *pde6b* mutation [32]. There are no reports of patients or mouse models with co-existing *pde6b* and *ush2a* gene mutations.

Because Kuming strain mice species background may interfere with the identification of auditory phenotype, we crossed the KM^ush/ush^ mice with CBA/CaJ, a strain with ‘‘gold standard” normal hearing [33, 34]. The F1 hybrids had normal ERGs and ABR thresholds. The phenotypes of F2 hybrids segregated into four types; of which 32 (16 males) presented elevated ABR thresholds, nine (2 males) presented declined ERG amplitudes, nine (2 males) presented both declined ERG amplitudes and elevated ABR thresholds and 63 (36 males) had normal ERG and ABR thresholds. These results indicated that KM^ush/ush^ mice were homozygous for separate mutations related to *pde6b* and *ush2a* and in autosomal recessive inheritance. Furthermore, we sequenced the coding region of the *pde6b* and *ush2a* genes and found that KM^ush/ush^ mice carried the rd1 allele, and the *ush2a* gene had 25 point mutations in 13 exons (unpublished). This could explain why the retinal degeneration of KM^ush/ush^ mice resembled that of rd1 mice. The mechanism of hearing loss and the reason why the expression of ush2a mRNA declined is not known.

In conclusion, our study demonstrated the characteristics of KM^ush/ush^ mice. This spontaneous hereditary blindness/deafness mouse model helped us understand the heterogeneity of RP and USH, and may provide insights related to the genotype-phenotype correlations of these two diseases. Future studies using this model may contribute to the understanding of the mechanisms associated with sensory development in general, and dual sensory loss in particular, and facilitate the development of therapies that prevent retinal degeneration in patients with Usher syndrome.

## Supporting Information

S1 File Overview of raw data. (ZIP)
